# A Radio-Fluorogenic Polymer-Gel Makes Fixed Fluorescent Images of Complex Radiation Fields

**DOI:** 10.3390/polym10060685

**Published:** 2018-06-20

**Authors:** John M. Warman, Matthijs P. de Haas, Leonard H. Luthjens, Antonia G. Denkova, Tiantian Yao

**Affiliations:** Department of Radiation Science and Technology, Faculty of Applied Sciences, Delft University of Technology, Mekelweg 15, 2629 JB Delft, The Netherlands; m.p.dehaas@tudelft.nl (M.P.d.H.); l.h.luthjens@tudelft.nl (L.H.L.); a.g.denkova@tudelft.nl (A.G.D.); t.yao@tudelft.nl (T.Y.)

**Keywords:** 3D dose imaging, radio-fluorogenic gel, polymer gel dosimetry, radio-fluorogenic co-polymerization, tertiary-butyl acrylate gel, proton beam imaging

## Abstract

We review the development and application of an organic polymer-gel capable of producing fixed, three-dimensional fluorescent images of complex radiation fields. The gel consists for more than 99% of γ-ray-polymerized (~15% conversion) tertiary-butyl acrylate (TBA) containing ~100 ppm of a fluorogenic compound, e.g., maleimido-pyrene (MPy). The radio-fluorogenic effect depends on copolymerization of the MPy into growing chains of TBA on radiation-induced polymerization. This converts the maleimido residue, which quenches the pyrene fluorescence, into a succinimido moeity (SPy), which does not. The intensity of the fluorescence is proportional to the yield of free-radicals formed and hence to the local dose deposited. Because the SPy moieties are built into the polymer network, the image is fixed. The method of preparing the gel and imaging the radiation-induced fluorescence are presented and discussed. The effect is illustrated with fluorescent images of the energy deposited in the gel by beams of X-rays, electrons, and protons as well as a radioactive isotope.

## 1. Historical Background

**Figure 1:**
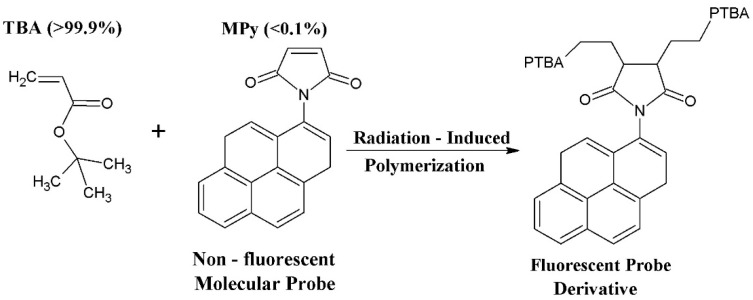
A schematic of the reaction mechanism underlying the radio-fluorogenic effect.

The development of radio-fluorogenic gels began with the demonstration by Warman et al. in 1997 that a dilute solution of the non-fluorescent molecule maleimido-fluoroprobe (MFP) in methyl methacrylate (MMA) became fluorescent on γ-irradiation [1]. The fluorescent product was found in the polymeric product fraction of the radiolysis and was therefore attributed to the co-polymerization of MFP into the growing PMMA chains. *Co*-polymerization of MFP results in the conversion of the fluorescence-quenching maleimido group into the succinimido group which does not quench the fluoroprobe fluorescence. A schematic of the basic process is shown in Figure 1 with the bulk monomer tertiary-buty acrylate and the fluorogenic compound maleimido-pyrene in this case.

This phenomenon was subsequently used to monitor the degree of monomer conversion of methyl methacrylate during the “Gel” or “Trommsdorff” effect in which autoacceleration of polymerization occurs due to the increasing viscosity of the polymerizing system slowing down termination [2,3,4,5,6,7,8].

**Figure 2:**
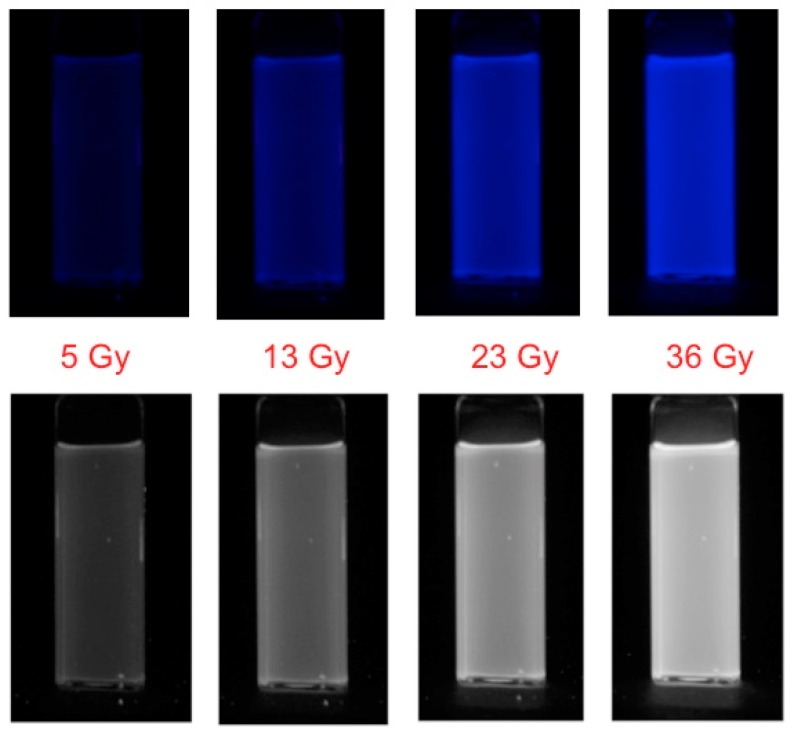
**Upper**: The full RGB color fluorescence in UV light of a 10 × 10 mm^2^ cuvette containing a 1.4 mM solution of maleimido-pyrene in tertiary-butyl acrylate, as a function of accumulated dose in a GC200 γ-ray source (dose rate ~2 Gy/min). **Lower**: The same cuvette with the fluorescence shown as blue-pixel gray-scale values.

Several years later, the present authors decided to investigate the possibility of using the radio-fluorogenic *co*-polymerization (RFCP) effect as a fluorescent method of dosimetry. To illustrate this, Figure 2 shows the increase in the fluorescence of a solution of maleimido-pyrene (MPy) in tertiary-butyl acrylate (TBA) with increasing accumulated dose.

Initially PTFE-encapsulated, small-volume (~0.2 mL) solutions of MMA were studied at ^60^Co γ-ray doses in the “linear” region, i.e., below ~20% monomer conversion. The fluorogenic molecule chosen for these studies was maleimido-pyrene (MPy) because of its commercial availability from Sigma-Aldrich (Zwijndrecht, The Netherlands) and its reasonably inexpensive price of ~200 euro per gram. Tertiary-butyl acrylate (TBA) was chosen in place of MMA as the bulk polymerizable monomer because of the larger propagation rate of acrylates compared with methacrylates [9,10]. This resulted in a close to 30-fold increase in sensitivity [11].

Even prior to these small-volume measurements, the idea had occurred to us that if the fluorescent image could be fixed in space it could provide a method for three-dimensional dosimetry. The problem at the time was finding a suitable bulk medium capable of immobilizing the fluorescent radiolytic product but still allowing polymerization to take place. The breakthrough came serendipitously in 2008 when we discovered that TBA itself formed a quasi-rigid gel when irradiated to a monomer conversion of ~15%, corresponding to a γ-ray dose of approximately 10 Gy [12,13].

The idea of using a gel medium containing a radiation-sensitive compound for 3D dose imaging was in fact first suggested by Day and Stein as long ago as 1950 [14]. Unknown to us at the time, several other groups had been working on potential polymer gel dosimetry systems [15] and a biennial conference, “DOSGEL”, which was specifically focused on 3D gel dosimetry, had been in existence since 1999 [16]. Rather than fluorescence however, the other methods being investigated used radiation-induced changes in optical absorption, nuclear spin relaxation, or turbidity as dose monitors [15].

**Figure 3:**
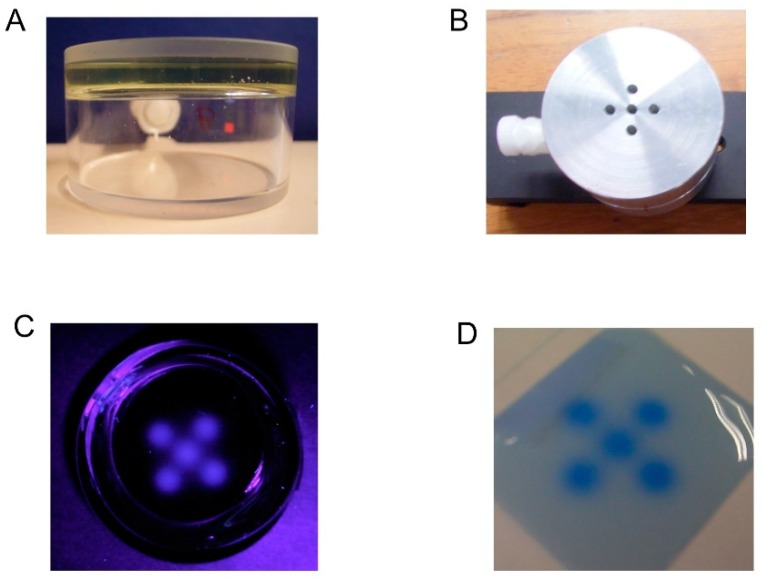
The first test measurement of fixed fluorescence imaging using the RFG gel production procedure described in the text. (**A**) A cylindrical spectrosil cell containing a (slightly yellow) 5 mm layer of a quasi-rigid gel containing ~1 mM MPy in TBA/15%PTBA. (**B**) The 5 mm thick aluminum mask for collimating the 3 MeV electron beam. (**C**) The fluorescent image of the gel in 365 nm light after irradiation with a dose of 20 Gy. (**D**) Comparison with a radio-chromic film irradiated under the mask to 10 Gy.

The answer to producing a radio-fluorogenic (RFG) gel would appear at first sight to be simple; irradiate an MPy solution in TBA to ~15% monomer conversion. The problem with this approach is that it produces a gel that already has a large background fluorescence that emanates from the whole medium. This significantly reduces the dynamic range of the sensitivity and the spatial resolution. We decided therefore to try a more complex procedure in which pure de-aerated TBA was first polymerized to ~15% in the container to be used in the imaging measurements. The remaining monomer was then removed by evacuation at room temperature and replaced by a de-aerated dilute solution of MPy in TBA. It was hoped that the polymer network would then swell over several days to give an RFG gel with a non-fluorescent background. This procedure was first put to the test in August 2008 using our in-house 3 MeV Van de Graff electron accelerator with a five-hole beam collimator covering the gel, as shown in Figure 3.

The well-defined fluorescent image of the five 2 mm beams on a black (non-fluorescent) background and the fact that the image faded only slightly over several days provided confirmation that the method did work.

These initial results were reported in an oral presentation at the DOSGEL meeting in 2008 but were too late to be included in the conference paper [17]. They have, in fact, never been published until now. That is also the case for preliminary results obtained in November 2008 in collaboration with Yves De Deene using the multi-leaf-collimated beam of a 6 MV linac in Gent, Belgium.

In the following sections, we will provide more details of the gel preparation procedure and properties, information on the imaging set-up used, and present images of a variety of radiation fields that have been investigated. The results have been reported to a greater or lesser extent in a variety of media outlets. They are collated in this review to illustrate the versatility of the method and the variety of areas in which high 3D spatial resolution dose imaging could be useful.

The practical area in which 3D dose imaging could be most useful is radiotherapy. Modern radiotherapy treatments of cancer involve the use of increasingly complex radiation fields that are adjusted to ensure maximum damage to the site of the malignancy with minimal collateral damage to neighboring healthy tissue. The need for a reproducible and readily available method of 3D dosimetry with high spatial resolution and rapid data analysis is clear [18,19,20]. Areas that would benefit from an accurate, fast-feedback measure of the actual dose delivered to a 3D phantom are; system commissioning, validation of computer protocols, tests of robotic functions, and personnel training. Pseudo-3D dosimetric methods based on physical arrays of single-point (e.g., ionization chamber) or 2D (e.g., radio-chromic film) detectors are at present not capable of providing the submillimeter spatial resolution required.

Unfortunately, there is still no generally accepted method of 3D dose imaging based on the various polymer gel methods that have been proposed over the years [15,19]. Two main barriers to acceptance have been the complexity, and resulting irreproducibility, of the gel formulations, and the lack of the ready availability of rapid, in-house image analysis. The method we present here, which has not been included in previous reviews, does not suffer from these particular limitations; the medium consists for more than 99% of a single component with a milli-molar concentration of an added fluorogenic compound whose concentration can be accurately determined spectro-photometrically. In addition, imaging and data analysis can be carried out on-the-spot within a few minutes of irradiation using a relatively simple, inexpensive set-up. The possibility of making 3D video images of complex fields has also been realized recently [21,22].

## 2. Materials and Methods

### 2.1. RFG Gel Preparation

**Figure 4:**
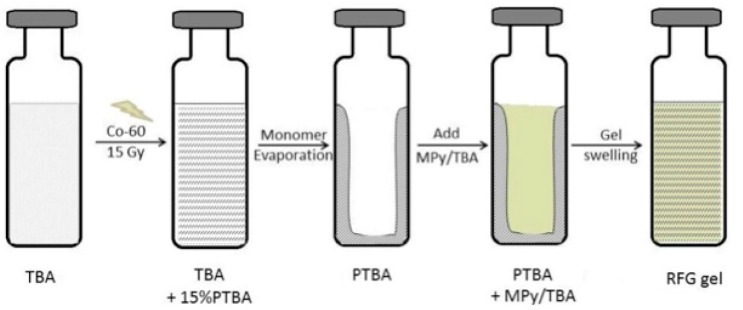
A pictorial representation of the processes involved in the preparation of a reformed radio-fluorogenic (RFG) gel from tertiary-butyl acrylate (TBA) and maleimido-pyrene (MPy).

Essential for the production of a radio-fluorogenic (RFG) gel is a nitrogen-flushed glove box. Its availability is assumed in the following description of the preparation procedure that is described below and illustrated in Figure 4. The gel does however not necessarily have to be produced on site.

Our present procedure is the following: (1) radiation-induced polymerization of de-aerated, inhibitor-free tertiary-butyl acrylate (TBA, purum from Sigma-Aldrich, Zwijndrecht, The Netherlands #327182) to ~15% monomer conversion using a large cavity ^60^Co γ-ray source (GC200 from Atomic Energy of Canada, Ottawa, ON, Canada) with a homogeneous dose rate of ~2 Gy/min. At this conversion the gel displays no tendency to flow over a period of at least an hour [13]. (2) Removal of the remaining monomer TBA by evacuation in a vacuum oven at room temperature for approximately one week. (3) Replacement of the monomer with a de-aerated TBA solution containing milli-molar of the fluorogenic compound maleimido-pyrene (MPy, Sigma-Aldrich P7908). (4) Regeneration of the gel on standing for approximately two weeks.

The reformed gel is clear, slightly yellow in color and is non- (or very weakly)-fluorescent. The concentration of MPy in the gel can be accurately determined from its optical absorption using an extinction coefficient of MPy at 365 nm of 790 L/mol/cm. Important for applications in radiotherapy is the tissue (or water) equivalence of the gel. The gravimetric density and electron density of 0.91 kg·L^−1^ and 3.00 × 10^26^ L^−1^ compare well with water, 1.0 kg·L^−1^ and 3.35 × 10^26^ L^−1^, and the frequently used ‘solid water’ substitute PMMA, 1.18 kg·L^−1^ and 3.84 × 10^26^ L^−1^.

In the absence of a glove box and/or homogeneous radiation facility at the place of measurement, the gel must be pre-prepared and transported to the site. This was the case for all of the away-from-home measurements presented in subsequent sections. More details of the gel preparation and properties are given in references [12,13].

### 2.2. Fluorescence Imaging

**Figure 5:**
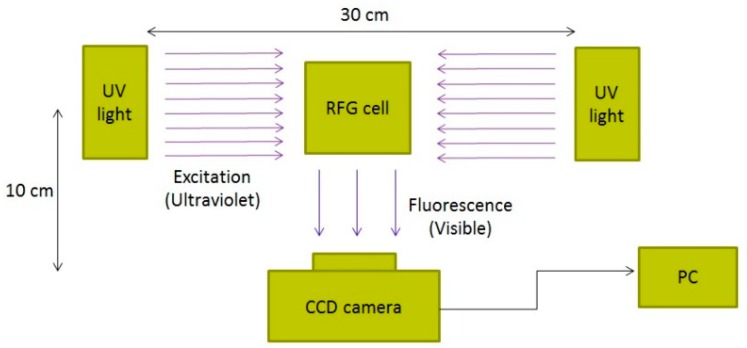
A schematic representation of the set-up used for fluorescence imaging of cells containing an irradiated RFG gel.

For imaging purposes, the gels in the measurements presented here were contained in cells that had optically flat sides; either cylindrical cells with optically flat end-walls or flat-wall, square cells with inner dimensions up to 4 × 4 cm^2^. The basic set-up is shown schematically in Figure 5.

Initially, mercury arc lamps with a Wood’s filter envelope (main emission 365 nm) were used. These have been replaced in more recent measurements by linear, multiple LED arrays with collimating optics [21,22]. The camera used for the earliest measurements reported was a RICOH Caplio RX which has been replaced by a RICOH GX200 that produces raw DNG and JPEG image files. In the DNG files, the pixel magnitude is linearly dependent on light intensity, in the JPG files the level is adjusted to give a ‘realistic’ visual representation of the image (see Figure 3.10 in reference [21]). The files were imported into ImageJ (freely downloadable from the National Institutes of Health [23]) for color separation and pixel level scanning. The spatial resolution within the gel is dependent on the camera settings but was normally better than a 0.1 mm per pixel. The images presented in the next section are of bulk gels and are shown simply to illustrate how the method can be adapted to the study of a variety of radiation sources. The images are not strictly 3D but we have recently developed a tomographic method of scanning the gels which allows us to produce full 3D video images [21,22].

In some figures, full-color RGB images of the fluorescence are given, in others grayscale values of the blue pixels are displayed since this corresponds to the wavelength region of maximum fluorescence emission i.e., ~400 nm.

### 2.3. The Radiation Sources

Initial experiments were carried out using the Reactor Institute Delft’s own Gammacell 200 ^60^Co γ-ray source (Atomic Energy of Canada) for gel preparation and calibration; the 3 MeV Van de Graaff electron accelerator (High Voltage Engineering) for the initial tests of fixed imaging, and the 250 kVp X-ray source (Philips MCN 321, Philips B.V., The Netherlands) for photon beam studies.

Other measurements have been carried out using the Elekta “Synergy” 6 MV radiotherapy treatment source at the University Hospital Gent, Belgium; The HDR brachytherapy treatment facility, and the “small animal” microbeam source at the Maastro Clinic, Maastricht, The Netherlands; The proton cyclotron beam source at the Centre for Advanced Radiation Technology (KVI), Groningen, The Netherlands; The “small animal” microbeam source at The Netherlands Cancer Institute (NKI), Amsterdam, The Netherlands; The Siemens Gammatron-3 ^60^Co γ-ray source at the Dutch National Metrology Institute (VSL), Delft, The Netherlands. Not all of these measurements have been published, but the names of all of the collaborating scientists are given in the acknowledgements.

## 3. Images of Various Radiation Fields

### 3.1. Homogeneous Gamma-Rays

In Figure 2 images are shown of the increase of the fluorescence intensity of an RFG gel with accumulated dose in a cobalt-60 γ-ray source. The gel sample was placed centrally in the 140 mm high, 90 mm diameter cylindrical cavity of the source. Over the 10 × 10 × 35 mm^3^ volume of the gel the dose rate within the GC200 cavity varied by less than 2% [24]. Rather than a linear or sub-linear dependence on dose, the results actually display a super-linear dose dependence of the fluorescence intensity [12,21,25]. This is also found for the dose dependence of monomer conversion in pure TBA [13] and is attributed to a decrease in the rate of radical-radical recombination as the viscosity of the medium increases.

### 3.2. MeV Electron Beam

**Figure 6:**
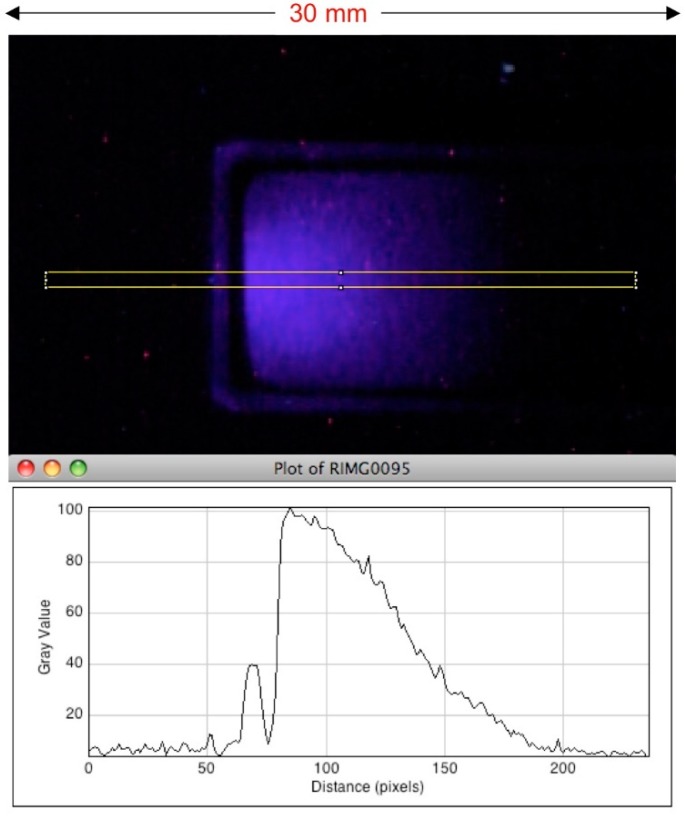
**Upper**: The depth-intensity image of the fluorescence of an RFG gel irradiated with a 3 mm diameter 3 MeV electron beam from a Van de Graaff accelerator. The yellow rectangle defines the limits of the plot-profile z-axis scan of the intensity shown in the **lower** part of the Figure. The pixel resolution was 0.11 mm per pixel. Taken from reference [26] Figure 9.

As mentioned in Section 1, the first test of an RFG gel for producing a fixed fluorescent image of a high-energy radiation field was carried out using the institute’s 3 MeV electron accelerator with an aluminum beam-mask, see Figure 3. Further e-beam experiments were carried out using a 10 × 10 mm^2^ cuvette with a single 3 mm diameter beam collimator fitted to its bottom. This allowed the image shown in Figure 6 to be made of the depth-intensity distribution along the propagation (Z) direction of the beam [26]. The form of the depth dependence is very close to that expected from the generalized depth–dose dependence for high-energy electrons in a medium of density ~0.9 kg/L [27].

The FWHM width of the beam could also be measured and increased from 5.5 to 8.1 mm in going from a depth in the gel of 0.5 to 5.4 mm [26]. This is characteristic of the large degree of scattering of (low particle mass) electron beams.

### 3.3. X-ray Beams

**Figure 7:**
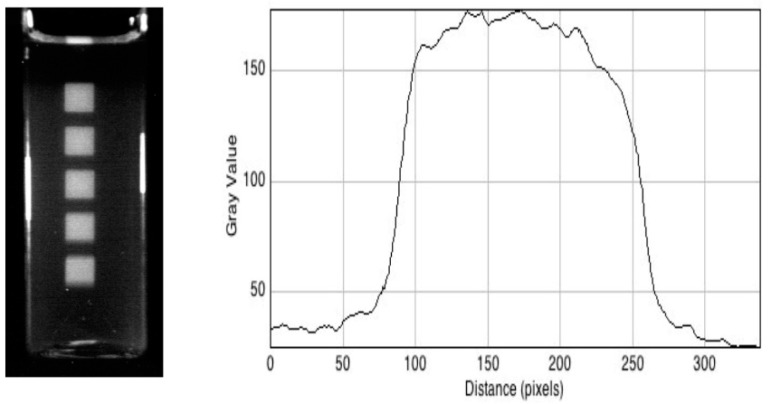
Left: A 20 mm square cell containing a 60 mm length of an RFG gel irradiated five times with a 5 mm square X-ray beam (dose 10 Gy) with the beams vertically displaced by 10 mm between irradiations. Right: A pixel profile scan across the central beam at 0.030 mm per pixel.

Images of high-energy photon beams were first made using the institute’s 250 kVp 15 mA X-ray source [12]. Figure 7 shows a 60 mm long RFG gel in a 20 mm square cell that has been irradiated 5 times with an X-ray beam collimated by a 5 × 5 mm^2^ square aperture in a 5 mm thick lead attenuator. The cell was displaced vertically by 10 mm between irradiations. A pixel-profile scan across the middle beam image is also shown in Figure 7. From this, the 20–80% rise and fall (the ‘penumbra’) is found to be 0.6 ± 0.2 mm. This is considerably larger than the pixel resolution of 0.030 mm/pixel and is ascribed to edge-dispersion in the X-ray beam.

**Figure 8:**
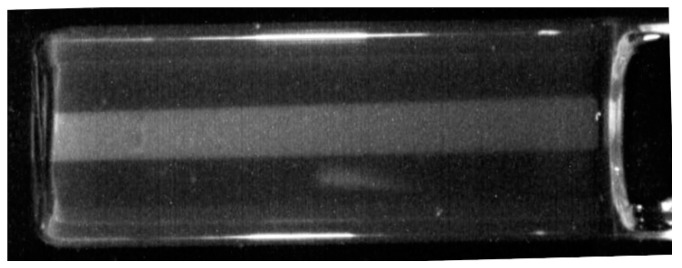
The image of a 5 × 5 mm^2^ 200 kVp X-ray beam propagating in a 60 mm length of RFG gel contained in a 20 × 20 mm^2^ cell. Optical artifacts occur at the glass/air sides of the cell and at the gel meniscus.

Figure 8 shows the image of a single 5 mm beam propagating along the length of a 60 mm long RFG gel. The gradual decrease in intensity over the length of the gel can be seen and is characteristic of the gradual attenuation of high-energy photons. The decrease in intensity was found to agree with the depth–dose measurements for 200 kVp X-rays made in water by Gerig et al. [12,28].

**Figure 9:**
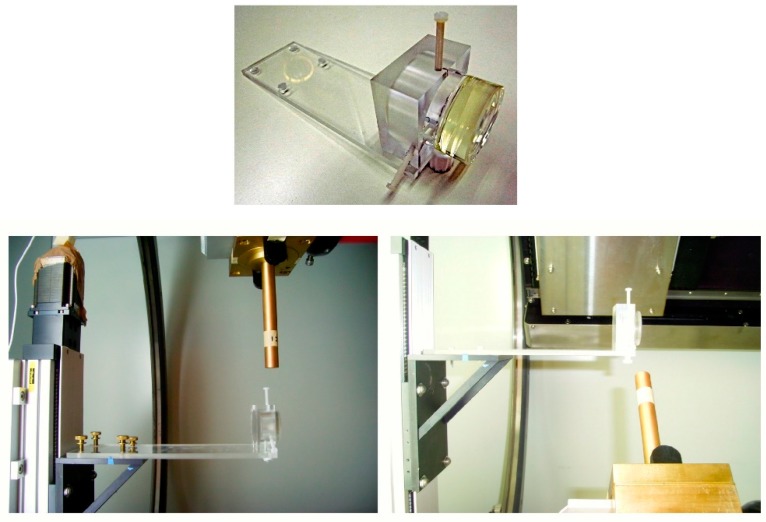
**Upper**: the PMMA RFG cell holder with a 20 mm thick RFG gel in a 50 mm diameter quarts cell for measurements in a “small animal” microbeam X-ray irradiation facility at the Antonie van Leeuwenhoek Ziekenhuis (Nederlands Cancer Institute) Amsterdam. **Lower**: a 6 mm gel positioned under the beam collimator, which could be rotated over 360 degrees in the X/Y plane.

Measurements were also carried out with the microbeam X-ray machines in Maastricht and Amsterdam which are mainly intended for small-animal investigations. A special PMMA cell holder was designed for this purpose and is shown in Figure 9.

**Figure 10:**
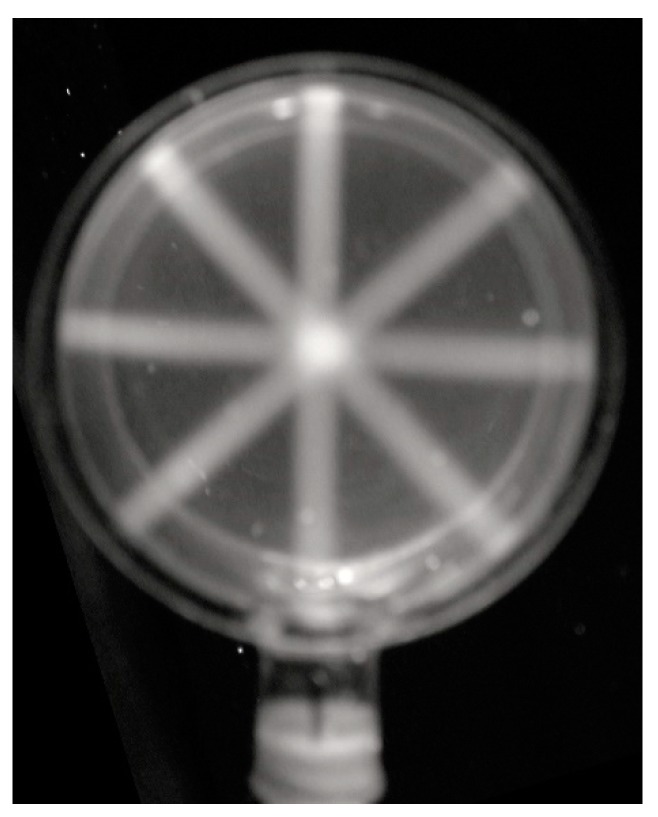
The fluorescence of a 6 mm thick RFG gel in a 50 mm diameter cylindrical quartz cell after irradiation with a 2.5 mm collimated, 250 kVp X-ray beam at 0, 45, 90, and 135 degrees to the vertical.

This apparatus could be used to carry out a very simple simulation of an intensity-modulated radiation therapy (IMRT) treatment procedure with multi-aspect beam irradiation. This is shown in Figure 10 where the gel has been irradiated with four 2.5 mm circular beams at angles of 0, 45, 90, and 135 degrees to the vertical. The much higher fluorescence intensity (dose) at the region of intersection of the beams is clearly visible.

**Figure 11:**
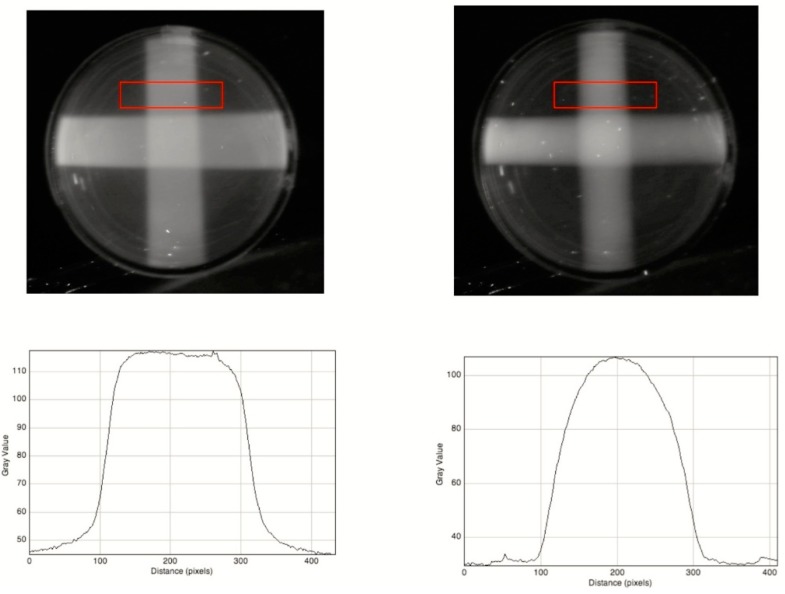
A 20 mm thick RFG gel irradiated with orthogonally-crossed X-ray beams. **Upper left**: a 10 × 10 mm square beam, **Upper right**: a 10 mm diameter round beam. **Lower**: pixel profiles across the beams as shown by the red rectangles. Pixel resolution 0.049 mm/pixel.

The different cross-sectional images of a 10 mm square and a 10 mm diameter round beam are illustrated in Figure 11. The pixel profiles show the difference between the top-hat shape for the former and the bell-shape for the latter. This illustrates that there is 3D information even in bulk images.

### 3.4. Proton Beams

**Figure 12:**
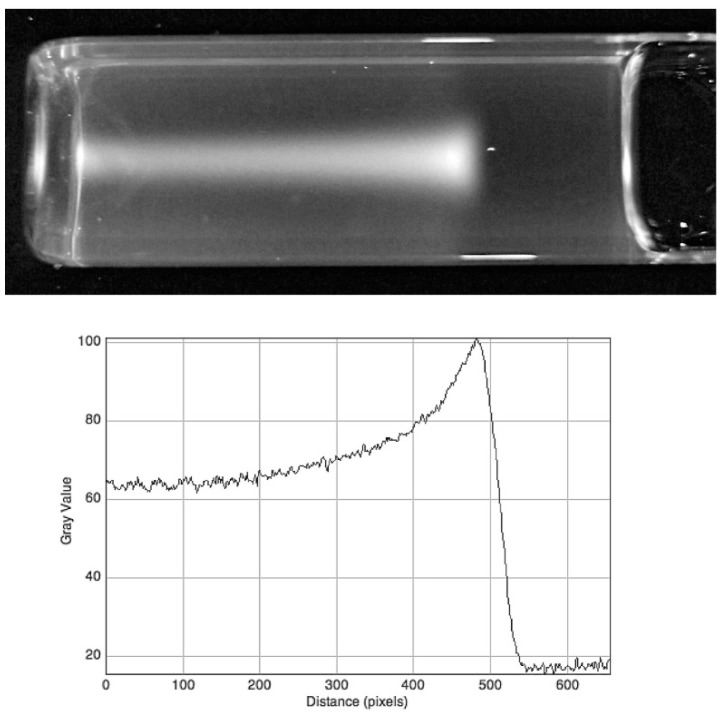
**Upper**: A grayscale image of an 80 MeV proton beam propagating in an RFG gel. **Lower**: A pixel-intensity profile along the axis of the beam; 0.061 mm per pixel.

The complete contrast between energy deposition by photon and proton beams (or particle beams in general) is illustrated by the difference between the X-ray image in Figure 8 and the image of an 80 MeV proton beam propagating in an RFG gel shown in Figure 12 [29].

The divergence of the beam just prior to the Bragg-peak cut-off is clearly visible in the upper image in Figure 12 as is the sharp cut-off just after the peak. The 80–20% cut-off occurs over a distance of 1.4 mm, which is to be compared with the total length of the track in the gel of 39 mm.

An aspect of considerable importance and discussion is the magnitude of the increase in dose rate in the region of the Bragg peak. The increase found in Figure 12 is much less pronounced than that found in Monte Carlo calculations or using ionization chambers. This tends to be a general finding of a difference between chemical and physical measurements of the phenomenon. In the present measurements, there are reasons why the magnitude of the pixel levels are not a quantitative measure of the local dose deposited. Firstly, the data shown are from JPEG files which provide a good visible representation of the fluorescence but are not a linear function of the intensity. Secondly, there are accumulated dose and dose rate dependences that have to be taken into account [21]. On the other hand, the present measurements do reflect chemical change in a condensed medium which may be more relevant to radio-biological effects than gas phase ionization.

**Figure 13:**
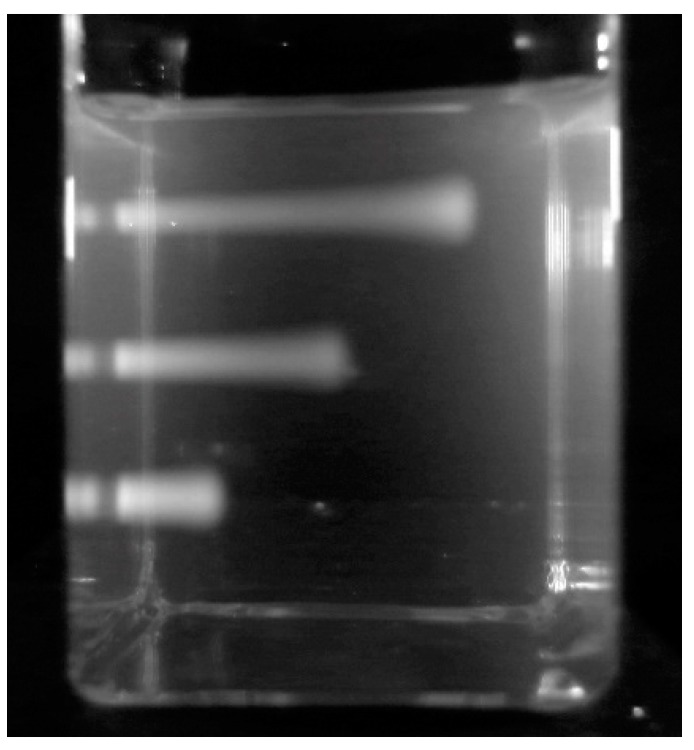
80 MeV proton beam tracks imaged in a 40 × 40 × 40 mm^3^ RFG gel; attenuated, from top to bottom, by polystyrene sheets 22, 32, and 42 mm thick. Portal dose 15 Gy.

A particularly important aspect of particle irradiation is that the position of the Bragg peak can be manipulated not only in the X/Y plane but also along the propagation, Z axis, by interposing a moderator in the beam. This is illustrated in Figure 13 by the decrease in range of the beam with increase in the thicknesses of polystyrene sheets interposed. The ranges in the gel (to 90% distal intensity of the Bragg peak) for 22, 32, and 42 mm polystyrene are 31.4, 20.2, and 8.6 mm. From this the water equivalent thickness (the WET value) of the gel is determined to be 0.91 [29].

### 3.5. “High Dose Rate” ^192^Ir Seed

**Figure 14:**
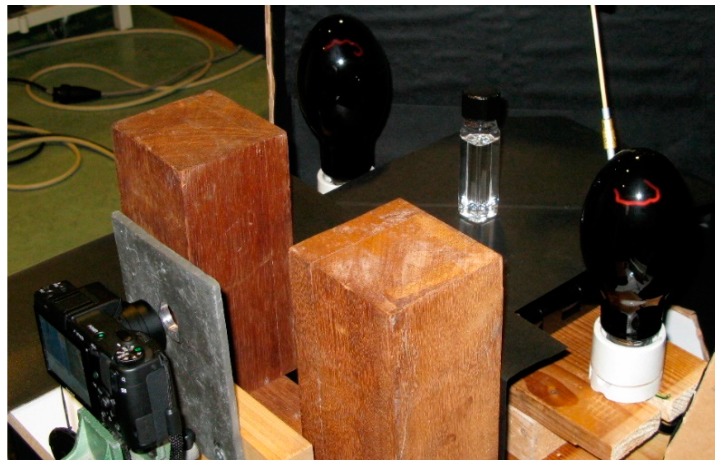
The set-up used to monitor, in situ, the fluorescence of an RFG gel on insertion of a seed of ^192^Ir. The main constituents are a 20 × 20 mm^2^ glass cell containing the gel into which a 1.9 mm diameter catheter (shown in Figure 15) is inserted via a septum cap; two mercury-arc UV (365 nm) lamps, a Ricoh GX200 digital camera with a lead shield against gamma irradiation and wooden shields against direct UV.

**Figure 15:**
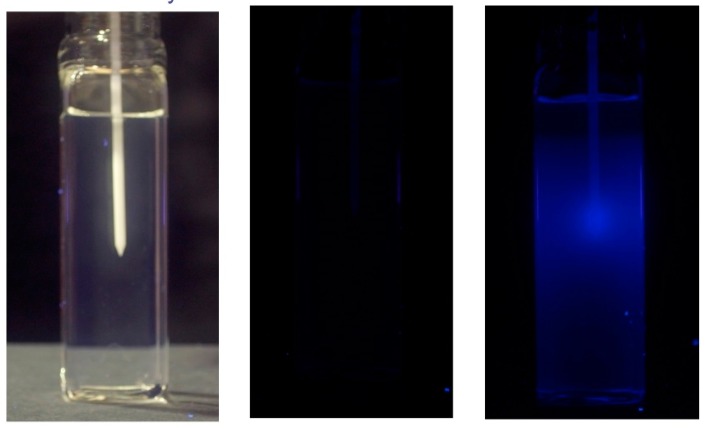
A 20 × 20 mm^2^ square, 60 mm long RFG gel with a catheter inserted via a septum seal for insertion of a high dose rate seed of Iridium-192. **Left**: In room lighting prior to seed loading. **Middle**: In UV light prior to seed insertion. **Right**: In UV light 3 minutes after seed insertion.

Possibly the most difficult spatial dosimetry problem in radiotherapy is that occurring in brachytherapy where the dose to the patient is dependent on the geometry of the isotopic seed, the encapsulation and the matrix of the multi-seed positions. This is further complicated by the extremely rapid (~1/*r*^2^) decrease in dose with distance from a seed. We devised a simple system for monitoring in situ the fluorescence of an RFG gel on introduction of a seed of ^192^Ir [30]. This set-up is shown in Figure 14. Insertion of the 3.6 mm long, 0.9 mm diameter seed into the catheter (shown in Figure 15) was accomplished using a Nucleotronics afterloader.

Figure 15 shows images of the cell and catheter taken before insertion of the seed in room lighting and after a residence time of 3 min. Images were in fact taken while the seed was still present at intervals of 10 s and a time-lapse movie has been produced that illustrates the gradual increase in the fluorescence with time [30]. Of potential interest is that images could also be made after retraction of the catheter and relaxation of the surrounding gel.

**Figure 16:**
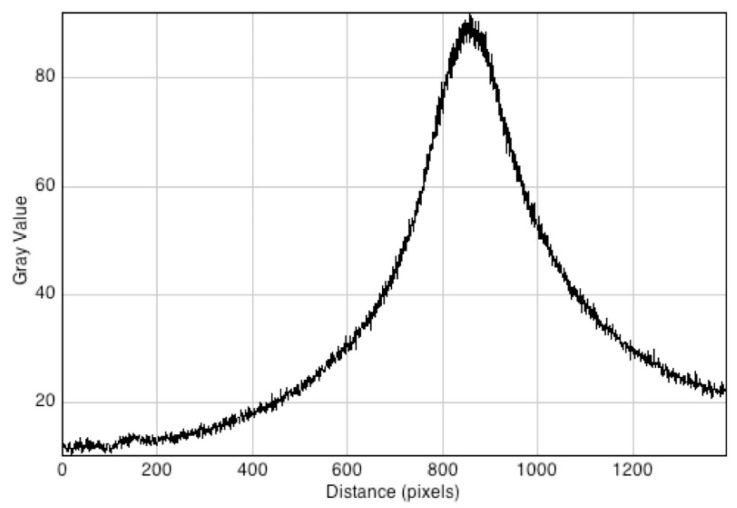
A pixel profile scan taken along the axis of the catheter, i.e., the centralvertical axis in Figure 15. The pixel resolution is 0.035 mm/pixel with a total scan length of approximately 50 mm.

The extremely rapid decrease of the dose (fluorescence intensity) with distance is shown by the pixel profile taken along the axis of the catheter to the bottom of the gel in Figure 16. From the pixel resolution of 0.035 mm/pixel, the decrease to half of the maximum value is 0.9 mm.

## 4. Conclusions

In this review, we have described the development of a radio-fluorogenic (RFG) gel medium that is capable of making fixed fluorescent images of complex radiation fields with submillimeter spatial resolution. Examples have been given of a variety of fields produced by different radiation sources together with the necessary adaptations of the cell design and method of measurement. All of the cases presented concern images of the bulk gel medium and are not strictly three-dimensional. In further developments of the imaging technique, we have constructed recently a prototype capable of producing 3D video displays and cross-sectional field analysis [21,22].

Based on this prototype, a user-friendly apparatus is being developed at the time of writing. The simplicity and non-toxic nature of the RFG gel formulation together with the availability of a readily transportable, turnkey imaging apparatus could possibly reduce the resistance to chemical methods of dosimetry in the radiotherapy clinic. We envisage application of such a tissue equivalent medium to the rapid control of radiotherapy treatment protocol software and hardware, and to the training of radiotherapy personnel. Application to other areas where high-energy radiation sources are used for materials processing or testing is also possible.
